# The mediating effect of geospatial thinking on the relationship between family capital and academic achievement in geography

**DOI:** 10.3389/fpsyg.2023.1067198

**Published:** 2023-02-17

**Authors:** Jianzhen Zhang, Ting Su, Xiaoyu Liang, Yanhua Xu, Ziyang Wang, Yuyao Yu, Jiahao Ge

**Affiliations:** ^1^College of Geography and Environmental Science, Zhejiang Normal University, Jinhua, Zhejiang, China; ^2^School of Geography and Environment, Jiangxi Normal University, Nanchang, Jiangxi, China; ^3^College of Education and Human Development, Zhejiang Normal University, Jinhua, Zhejiang, China

**Keywords:** family capital, academic achievement in geography, geospatial thinking, mediating effect, upper-secondary-school students

## Abstract

**Purpose:**

Family environment has the major impact on children’s academic development. The aim of this study was to research the relationship between family capital and academic achievement in geography. Further, geospatial thinking, as a form of spatial thinking focusing on the scale of the geographical environment, is closely related to family environment and academic achievement in geography. Thus, the study was more specifically to apply a mediation model to explore the potential mediating role of geospatial thinking.

**Methods:**

A total of 1,037 upper-secondary-school students in Western China were surveyed using t the *Family Capital Questionnaire* and the *Geospatial Thinking Test Questionnaire*. SPSS (version 26.0) was used for descriptive statistical analysis and correlation analysis. The PROCESS plug-in (version 4.0) was used to test the mediating effect of geospatial thinking.

**Results:**

(1) The correlation analysis showed that family capital has a positive effect on academic achievement in geography and is related to geospatial thinking. Moreover, geospatial thinking exerts a positive effect on academic achievement in geography. (2) The results of mediation analysis indicated that geospatial thinking plays mediating and buffering roles in the relationship between family capital and academic achievement in geography after controlling for family residence and gender. The direct and indirect effects accounted for 75.32% and 24.68% of the total effect, respectively.

**Conclusion:**

The results indicated that family capital not only affected academic achievement in geography directly but also indirectly through geospatial thinking. This finding provides some ideas for the development of geography education, which suggests that geography educators need to pay more attention to the influence of the family environment on students’ geography learning in curriculum design and teaching processes. Also, the mediating role of geospatial thinking further uncovers the mechanisms underlying the academic achievement in geography. Therefore, it is necessary to focus on both students’ family capital and geospatial thinking in the process of geography learning, and carry out more geospatial thinking training to improve academic achievement in geography.

## 1. Introduction

Geography education plays a key role in the Framework for 21st Century Learning (Trilling and Fadel, 2009), refer to the development of 21st Century Skills (Sugiyanto et al., 2018). Geography is increasingly recognized as a core subject, because of its relevance to students’ critical thinking and global awareness (Lambert and Jones, 2017). Academic achievement in geography has also received significant global attention as a part of the academic achievement of students in the curriculum. The National Assessment of Education Progress (NAEP) of the United States has repeatedly conducted geography assessments to evaluate trends in students’ academic achievement in geography (Solem et al., 2021), and increased academic achievement in geography helps develop students’ critical, creative thinking (Sugiyanto et al., 2021). In the GeoCapabilities project, sponsored by the European Union’s Comenius Program, academic achievement in geography is linked to imagination and reasoning skills (Lambert and Jones, 2017). In addition, geography education has been linked to the understanding of social and environmental issues, and a number of studies have shown that the development of academic achievement in geography helps students to understand global issues and cultivate human-environment thinking (Israel, 2012; Larsen et al., 2022).

However, there are a number of factors affecting students’ academic achievement in geography, which can be broadly classified into two categories: internal factors (individual student factors) and external factors (e.g., family, school, and social aspects). Research has shown that gender, attitudes to learning and health behaviors can affect academic achievement in geography (Escolano-Pérez and Bestué, 2021). At the same time, individual geospatial thinking is also considered to be an important element in the achievement of the geography profession (Huynh and Sharpe, 2013). Spatial thinking refers to the way of thinking about visualizing and solving problems in space (Nielsen et al., 2011). Geospatial thinking is specialized spatial thinking and has the characteristics of spatial thinking (Verma, 2014). Research found that students with strong geospatial thinking perform better in understanding geographic concepts and are more successful in their geography learning (Klonari and Likouri, 2015; Xie et al., 2022). In addition, the external environment in which students live has also been linked by researchers to academic achievement of geography. It has been found that classroom climate, teachers’ quality, and family educational expectations all influence students’ academic achievement in geography (An et al., 2019; Ozdemir and Ozturk, 2022).

Family is a necessary environment in children’s development and is considered to be an essential ingredient in the development of spatial thinking (Clingan-Siverly et al., 2021). For example, Potter et al. (2013) found that cultural capital in the family (e.g., parents’ educational expectations) influences children’s spatial thinking and the development of related neural networks. As a part of spatial thinking, geospatial thinking is characterized by spatial thinking (Huynh and Sharpe, 2013) and can also be influenced by family (Zhang et al., 2022). Also, family is considered to be a critical factor influencing the academic achievement in geography (An et al., 2019). For example, Solem et al. (2021) identified that the amount of books collected in the home and the education level of the parents predicted students’ geographic achievement. Bravo Sanzana et al. (2017) discovered that family cultural capital has an impact on children’s achievement acquisition in geography. However, existing research does not link family capital, which is defined as the combination of the family economic, cultural, and social capital, to geospatial thinking and academic achievement in geography.

Therefore, in order to clarify the relationship between family capital, geospatial thinking and academic achievement in geography, this study explores the connection between family capital and academic achievement in geography, along with the mediating role of geospatial thinking between the two. Also, the effects of gender and place of residence on academic achievement were controlled in the analysis. In the next section, the definitions of the three variables, the relevant theories, the influencing factors and the links between the variables are presented.

## 2. Theoretical basis and hypothesis

### 2.1. Family capital

Family capital is the sum of the resources held by the family, including the economic status, education level, occupation and so on. According to Bourdieu, capital includes economic, cultural and social capital (Bourdieu and Richardson, 1986). Families in different social classes have different capital characteristics that determine the academic achievement of their children (Bourdieu, 1973). Similarly, Coleman describes the main forms of family capital in financial, human and social terms (Coleman, 1990). Specifically, financial capital is the position of wealth in the family (e.g., family income, etc.) that helps to provide resources and opportunities for children’s education. The human capital of the family, including the educational level and cognitive status of the parents, helps to provide a cognitive environment conducive to the intellectual and thinking development of the offspring. Social capital refers to resources that can contribute to the development of children, where the relationship between children and parents is considered to be part of the family’s social capital and this relationship affects children’s growth (Coleman, 1988). In essence, the human, financial and social capital of parents continues to influence the academic status and future achievements of their children through the cultural transmission of habitus, economic resources and interpersonal relationships. Therefore, all three types of capital are vital to the family process over several generations.

Family capital is considered to be an important factor in the health, thinking and educational status of individuals (Weinberg et al., 2019; Wang and Huang, 2021). In terms of personal health, researchers have found that children with poorer family capital exhibit physical disorders, sleep problems (Bøe et al., 2012)and poorer health, and are also more likely to suffer from psychological disorders such as depression (Zhou, 2018). In terms of thinking skills, research has identified that children’s thinking and ability development is influenced by parent–child interactions (Biro et al., 2009; Yuan and Ngai, 2019). At the same time, children with rich family capital have higher levels of creativity, innovation and cognitive ability (Liu et al., 2020; Xu and Pang, 2020). In terms of educational status, Coleman notes that parents’ educational attainment, family book collection, etc., affect their children’s educational achievement (Coleman, 1968). Children with higher family social capital tend to achieve higher levels of educational attainment (Coleman, 1988). A possible explanation for this is that advantaged families use direct resources and indirect cultural transmission to turn family capital advantages into educational opportunity advantages, which influences individual academic achievement (Green et al., 2015). Conversely, children from less privileged families have less access to education, which is detrimental to cognitive development (Brooks-Gunn and Duncan, 1997; Von Stumm et al., 2022).

According to the existing literature, most evaluations of family capital similarly identify family income and parental education as key factors (Hanson and Chen, 2007; Xu and Pang, 2020). For example, De Pernillo et al. (2014) used the highest level of parental education, family income, etc., as a basis for judging family capital. The major international education assessment project (PISA) also uses parental education, income and material resources as indicators of family background (Wang and Huang, 2021). This study used the Family Capital survey questions in the PISA 2018 student questionnaire to collect information on the family background such as parents’ education level, occupation, family ownership (e.g., desk, dictionary, etc.) and family book collection.

### 2.2. Academic achievement in geography

The concept of academic achievement has a broad and a narrow meaning. In a broad sense, academic achievement includes students’ performance in terms of knowledge and skills (Mega et al., 2014). For example, countries such as the United States and Australia have conducted competency assessments to reflect students’ proficiency levels (Collie et al., 2015; Jones and Mueller, 2017). Academic achievement in a narrow sense refers to a students’ examination results (Kristjnsson et al., 2009). A large number of studies have used exam or test scores as a measure of academic achievement (Lüftenegger et al., 2016). The definition adopted in this study is the narrow one, using students’ performance on a geography exam as a criterion for determining academic achievement in geography.

There are series of factors that influence academic achievement in geography, which can be divided into internal and external factors. Internal factors include, for example, individuals’ gender, intelligence, attitude toward learning, etc. (Filgona and Sababa, 2017; Marciano and Camerini, 2021). Filgona and Sababa’s study (2017) indicated that girls performed better than boys in terms of geography. Gil-Espinosa et al. (2019) noted that academic achievement in geography was significantly, although weakly correlated with students’ intelligence. Similar studies have shown that students’ executive functioning, physical activity and gender have a positive impact on academic achievement in geography (Escolano-Pérez and Bestué, 2021). Similarly, students with a positive attitude to learning tend to do better in academic achievement in geography (Díaz-Serrano and Martínez, 2016). External factors refer to family, school, society, etc. Parental educational expectation is considered to be a factor influencing academic achievement in geography (Bravo Sanzana et al., 2017). Besides, there was a link between students’ academic achievement in geography, teacher-student relationships, educational philosophy and the qualifications of geography teachers (Filgona and Sakiyo, 2020; Ho, 2021). Ozdemir and Ozturk (2022) found that students performed better in geography and learning in VR settings. It has also found that social media like Facebook as a teaching tool can improve academic achievement in geography (Al Zboon et al., 2018).

Nevertheless, fewer studies have focused directly on the relationship between academic achievement in geography and family capital, although there is evidence that family capital affects students’ learning status in geography. Research has shown that students with better family capital have more opportunities to go on trips and expeditions (Chiu and Chow, 2015), and such field activities are considered to be valuable geography learning experiences that help to develop interest and geography skills (Rydant et al., 2010; Krakowka, 2012). Interestingly, the study of geography is a lifelong learning process. The more time you have been exposed to geography, the more likely you are to achieve a high level of academic achievement in geography (Downs, 2014). In contrast, children with advantaged family capital are more likely to be exposed to learning tools such as maps and globes both before and during the trip, which increases their exposure to geography and strengthens their understanding of geography knowledge (Bein et al., 2009).

Combined with the overview of family capital, we derive the following hypotheses:

*Hypothesis 1*: Family capital positively affects academic achievement in geography.

### 2.3. Geospatial thinking

Thinking is regarded as an advanced stage of human cognition and process (Zhang, 2002) and is closely related to daily life and learning (Liu et al., 2021). Spatial thinking, as a part of thinking, refers to a combination of an individual’s cognition, skills and performance (Lee and Bednarz, 2012) and emphasizes abstract comparison and analysis of things from a spatial perspective (Hespanha et al., 2009). Learning to Think Spatially, published by the National Academy of Sciences, suggests that spatial thinking is an organic combination of the nature of space, methods of representing spatial information and the process of spatial reasoning (National Research Council, 2006). Nielsen et al. (2011) defined it as a way of thinking about visualizing and solving problems spatially, which in this case includes all space from the microscopic to the planetary scale. Spatial thinking plays an indispensable role in life, scientific research and education. The study found that most people rely on spatial thinking to choose their travel routes and find what they need in shops (Hespanha et al., 2009). At the same time, spatial thinking help develop key competencies (e.g., the ability to think through solutions, make decisions) and performance in subjects such as science and astronomy (Favier and van der Schee, 2014; Cole et al., 2018).

Geospatial thinking has been regarded as a specialized form of spatial thinking (National Research Council, 2006). Scholars consider it as a form of spatial thinking focusing on the scale of the earth, landscape and environment (Bodzin et al., 2014). It is different from spatial thinking, covering all spatial scales, while geospatial thinking is mainly applied in analyzing problems at the earth scale and requires the use of geographical knowledge and technology (Huynh and Sharpe, 2013; Xie et al., 2021). Bednarz (2011)defined geospatial thinking as the knowledge, skills and thinking habits of solving problems by using geographical information (such as maps, etc.) and reasoning process in a specific environment. However, there is a lack of effective ways to assess it (Huynh and Sharpe, 2013). Initially, psychologists developed spatial thinking test questions to support the assessment of geospatial thinking (Kail et al., 1979). However, the tests developed by psychologists are not fully applicable to the evaluation of geospatial thinking (Lee and Bednarz, 2009). In 2003, Lee and Bednarz (2009) designed a test to measure geospatial thinking. They adapted and revised it to update the Geospatial Thinking Test (STAT) instrument in recent years (Lee and Bednarz, 2012). Since then, the test has been widely used (Collins, 2018). Consequently, the geospatial test questionnaire used in this study draws on the Spatial Thinking Aptitude Test (STAT) instrument developed by Lee and Bednarz (2009).

The factors that influence geospatial thinking are more complex. First, individual differences in intelligence, gender and learning ability can affect the level of geospatial thinking (Aliman et al., 2019; Xie et al., 2021). Some studies have shown that males achieve higher scores on geospatial tests than females (Shin et al., 2016). There is also evidence that no significant differences were revealed (Zhang et al., 2022). Collins (2018) stated that students’ academic achievement in geography is related to their level of geospatial thinking. Second, research in the field of brain science and neuroscience have identified the brain has special structures for processing spatial information, which consists of numerous neural systems (Ivanitskii et al., 2015; Demir-Lira et al., 2016), supporting the development of geospatial thinking. Third, environmental factors are also believed to be important in influencing geospatial thinking. One study found that geography textbooks contain review questions about geospatial thinking (Scholz et al., 2014), which can help to improve geospatial thinking. At the same time, the use of paper and digital teaching media is helpful to cultivate students’ geospatial thinking (Collins, 2018). Similarly, the use of web maps in the teaching and learning environment is considered to be an effective way to enhance students’ spatial thinking in geography (Manson et al., 2014).

There are fewer existing studies that focus on the relationship between family capital and geospatial thinking, but it is proved that families have an impact on students’ thinking skills and spatial performance. Researchers have found that the family environment is crucial for children’s development, fostering their neural networks, which contribute to the development of learning and academic skills (Potter et al., 2013; Clingan-Siverly et al., 2021). In addition, there are richer resources in advantaged families to help nurture children’s knowledge base and promote the development of thinking ability (Uhlenberg and Geiken, 2021). What’s more, well educated parents are willing to spend time on constructive activities with their children and use maps, spatial language, etc., in their interactions to promote children’s thinking development (Borriello and Liben, 2018). Based on this, the following hypothesis is proposed:

*Hypothesis 2*: Family capital positively affects geospatial thinking.

Geography is a subject related to space (IGU CGE, 2019) and geospatial thinking is a crucial thinking skill for learning it. Nazareth et al. (2019) argued that geospatial thinking occupies an imperative place in the professional field of geography. It is worth noting that spatial thinking underpins the practice and theory of geography (Huynh and Sharpe, 2013; Jo and Bednarz, 2014) and contributes to students’ performance in science, astronomy, etc. (Cole et al., 2018). As a result, scholars have worked to develop educators’ abilities to use geospatial technology, defined as a superset of technologies, such as GIS, RS, etc. (Metoyer and Bednarz, 2017). Educators are more willing to teach using spatial technology to promote spatial thinking and develop students’ geospatial thinking (Baker et al., 2015). For example, Carbonell-Carrera and Hess-Medler (2019) used GIS to teach geography in the classroom and found that students’ geospatial thinking was improved. Collins (2018) also found that teaching geography incorporating Google Earth software promoted students’ geospatial thinking. Similar studies have shown that geospatial thinking helps students understand geographical data and influences the acquisition of geospatial knowledge (Perugini and Bodzin, 2020). And beyond that, individuals with strong geospatial thinking tend to succeed in the study (Carbonell-Carrera et al., 2020). In general, students’ geospatial thinking can affect their academic achievement in geography (Aliman et al., 2019). Therefore, we contend as follows:

*Hypothesis 3*: Geospatial thinking has a positive predictive effect on academic achievement in geography.

Based on the literature and the three hypotheses above, we further propose the following hypotheses:

*Hypothesis 4*: Geospatial thinking acts as a mediator between family capital and academic achievement in geography.

**Figure 1:**
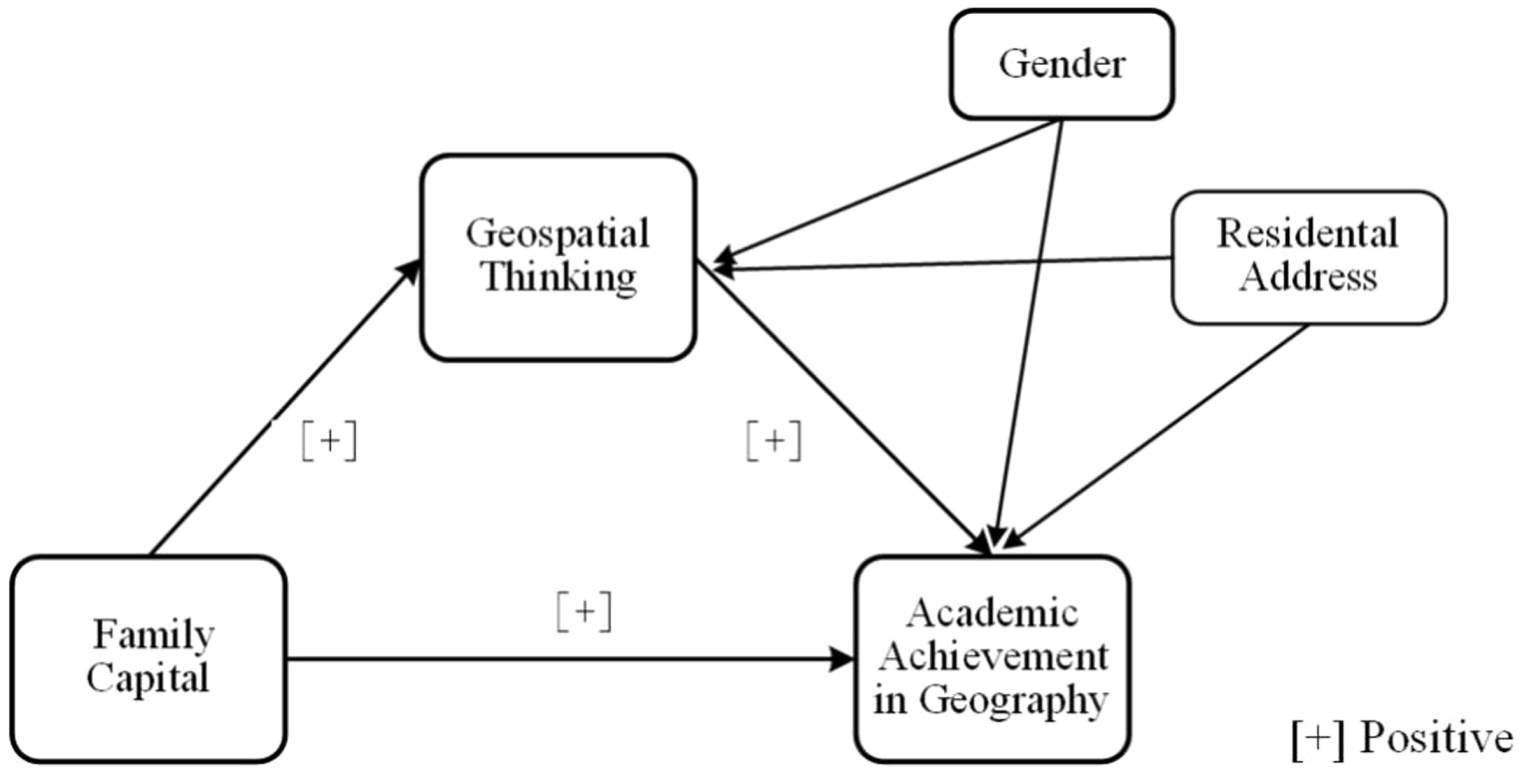
The relationships examined in the study.

Figure 1 shows a diagram of the mediation model proposed in the four hypotheses that depicts the relationships between the independent, mediator, and dependent variables and two covariates.

## 3. Materials and methods

### 3.1. Participants and procedures

Public secondary school students in a region of western China, between the ages of 16 and 18, were selected as participants in this study. The survey was conducted by distributing a paper questionnaire completed between 10 and 30 November 2021. First, before completing the questionnaire, the researcher explained the study and the details of the questionnaire to the participating students. Second, with the consent of parents, class teachers and the students themselves, the researcher distributed paper questionnaires to the students and asked them to fill them out truthfully. Finally, we collected the questionnaires and input the data.

After data collection was completed, the researchers tested the validity of the questionnaire and the actual number of valid questionnaires was 1,037. The statistical results are presented in Table 1. Among the interviewees, in terms of gender, 260 (25.0723%) were male and 777 (74.9277%) were female. In terms of residence, 621 (59.8843%) were urban, and 416 (40.1157%) were suburban. In addition, before the research design was finalized, the researchers conducted focal interviews with students about the relationship between family capital, geospatial thinking and academic achievement in geography. Many participants indicated that students with better family capital also had higher academic achievement in geography.

**Table 1:** Descriptive statistics for the three variables.

Variable		*N*	*M*	SD
Family capital		1,037	0.0004	1.8105
	*Gender*			
	Male	260	0.1425	1.9346
	Female	777	−0.0472	1.7658
	*Residential address*			
	Urban	621	0.7774	1.7376
	Suburban	416	−1.1594	1.1920
Academic achievement in geography		1,037	3.0700	1.1090
	*Gender*			
	Male	260	3.2500	1.2380
	Female	777	3.0100	1.0560
	*Residential address*			
	Urban	621	3.2500	1.1320
	Suburban	416	2.8000	1.0170
Geospatial thinking		1,037	8.4300	2.7080
	*Gender*			
	Male	260	8.5300	2.9710
	Female	777	8.3900	2.6160
	*Residential address*			
	Urban	621	8.8200	2.7550
	Suburban	416	7.8500	2.5310

### 3.2. Materials

The questionnaire used in this study consists of two parts and contains four items: demographic information, academic achievement in geography information, the *Family Capital Questionnaire* and the *Geospatial Thinking Test Questionnaire*. In the first part, demographic information was collected, including the gender and residence of the respondents. Second, with the consent of teachers, parents and students, the geographical learning achievements of the students were collected as a representative of academic achievement in geography. As this study was conducted at different school, the results of students’ geography examination were used as a criterion to judge their academic achievement in geography. The scores for each grade of students’ academic performance in geography were therefore divided into six levels: 90 and above (Level 6), 80–89 (Level 5), 70–79 (Level 4), 60–69 (Level 3), 40–59 (Level 2) and below 40 (Level 1). Besides, the researchers contacted geography teachers at the surveyed schools to ensure that the questionnaire was administered 1 week after the midterm exam to guarantee that participants could accurately recall their geography exam results. In addition, the data was rigorously screened by the researchers to eliminate questionable samples and to assure the validity of the sample data.

The second part includes the *Family Capital Questionnaire* and the *Geospatial Thinking Test Questionnaire*. The questionnaires used in this study were taken from the English version and were therefore back-translated to improve the quality of the translation (Brislin, 1970). Primarily, the first researcher translated the English questionnaire into Chinese. Then, the second researcher translated them into English. Finally, the third researcher compared the original (English), translated (Chinese) and back-translated versions (English) of the questionnaire to ensure consistency in the meaning expressed in the original English and the translated version. In addition, the questionnaire was adapted and optimized by the researchers before the final questionnaire was accomplished.

### 3.3. Family capital questionnaire

The *Family Capital Questionnaire* has been modified based on the Family Background Survey items from the PISA 2018 Student Questionnaire.^1^ The final questionnaire had six questions, including parental education, parental occupation and family ownership. First, the parents’ education represents the family cultural capital, ranging from 1 (primary school) to 7 (PhD). Second, the parents’ occupation represents the family’s social capital, ranging from 1 (government/authority cadre/civil servant) to 12 (other inconveniently classified occupations). Family ownership points reflect the family economic capital, with points awarded for owning a certain number of items, and no points awarded for not owning items. Then, the two variables were standardized according to existing studies. Second, standardized z-scores were included in the factor analysis (Pokropek et al., 2017). Finally, the total score was used as an indicator of family capital, with higher scores predicting higher levels of family capital.

### 3.4. Geospatial thinking test questionnaire

The *Geospatial Thinking Test questionnaire* draws on the Spatial Thinking Aptitude Test (STAT) instrument developed by Bednarz and Lee (2019)^2^. The questionnaire has 16 questions on directional discrimination, map reading and using, such as: “The closest option to the landform you see is,” “The one that fits the logical operation of the map is,” etc. Students’ ability of geospatial thinking is determined using a scoring system (1 mark for a correct answer, no mark for an incorrect answer) which means that score are positively correlated with geospatial thinking. In this study, the Cronbach’s alpha for the scale was 0.695, indicating that there is a good correlation between the items of the scale (De Vaus, 2002).

### 3.5. Data analysis

This study used SPSS 26.0 software and PROCESS 4.0 plug-in to analyze the data. First, a Harman one-way test was used to test for common method bias before processing the data to ensure the validity of the data analysis (Podsakoff et al., 2003). The results showed that a total of seven factors had eigenvalues greater than 1, with the first factor accounting for only 18.217%, much less than the 40% threshold, so the common method bias problem in this study was small(Li et al., 2020). Second, following the reliability and validity analysis, the mean and standard deviation of the data were calculated using SPSS software to test for trends in the concentration and dispersion of the study data. Then, Pearson correlation coefficients were calculated to test the relationship between family capital, academic achievement in geography and geospatial thinking. Finally, a mediation analysis using the PROCESS 4.0 plug-in in SPSS was performed to explore the mediating role of geospatial thinking and to test the four hypotheses of this study.

## 4. Results

### 4.1. Descriptive statistics and correlations analyses

The results of the descriptive analysis of family capital, academic achievement in geography and geospatial thinking are shown in Table 1. There is much wider variation in geospatial thinking scores than in both family capital values and academic achievement in geography.

Next, the variables were analyzed for correlation by calculating Pearson correlation coefficients. The results showed (see Table 2) that there was a positive correlation between the three variables. First, there was a significant positive relationship between upper-secondary-school students’ family capital and their academic achievement in geography (*r* = 0.3860, *p* < 0.001). Second, there was a significant positive correlation between family capital and geospatial thinking (*r* = 0.3640, *p* < 0.001). In addition, there was a significant positive correlation between geospatial thinking and academic achievement in geography (*r* = 0.3580, *p* < 0.001).

**Table 2:** Pearson’s *r* for the three variables.

Variables	Family capital	Academic achievement in geography	Geospatial thinking
Family capital	1		
Academic achievement in geography	0.3860***	1	
Geospatial thinking	0.3640***	0.3580***	1

***p < 0.001.

### 4.2. Mediation analysis

The final hypothesis of this study was to test the mediating role of geospatial thinking. Using the PROCESS plug-in in SPSS (version 4.0), a mediation analysis was conducted with family capital as the independent variable, academic achievement in geography as the dependent variable and geospatial thinking as the mediating variable(Model 4). Furthermore, based on the literature review, gender and household residence were used as control variables in this study, both of which were transformed into dummy variables before being entered into the mediation model.

The results showed (see Table 3) that family capital had a significant positive predictive effect on academic achievement in geography (*β* = 0.2350, *t* = 11.4040, *p* < 0.001), and the prediction remained significant even with the addition of geospatial thinking variable (*β* = 0.1170, *t* = 8.4150, *p* < 0.001). Moreover, family capital was a significant positive predictor of geospatial thinking (*β* = 0.5610, *t* = 11.0080, *p* < 0.001). There was also a significant positive predictive effect of geospatial thinking on academic achievement in geography (*β* = 0.1030, *t* = 8.4390, *p* < 0.001). In addition, both the direct effect of family capital on academic achievement in geography and the mediating effect of geospatial thinking had bootstrap confidence intervals(95%), with no zero between their lower and upper limits (see Table 4). It means that, after controlling for gender and household residence variables, family capital can predict academic achievement in geography directly, and through geospatial thinking indirectly. The direct effect (0.1770) and the indirect one (0.0580) accounted for 75.3191% and 24.6809% of the total effect, respectively.

**Table 3:** Results of mediation analysis for the observed variables.

Regression equation		Fitting indices				Significance
Outcome variables	Predictor variables	*R*	*R* ^2^	F(𝑑𝒇)	*β*	*T*
Geospatial thinking		0.3650	0.1330	52.7970***		
	Gender				−0.0360	−0.1970
	Residential Address				0.1170	0.6250
	Family capital				0.5610	11.0080***
Academic achievement in geography		0.4570	0.2090	68.2830***		
	Gender				−0.1950	−2.7580**
	Residential Address				−0.0050	−0.7100
	Geospatial thinking				0.1030	8.4390***
	Family capital				0.1170	8.4150***
Academic achievement in geography		0.3930	0.1550	63.0200***		
	Gender				−0.1990	−2.7190**
	Residential Address				0.0070	0.0900
	Family capital				0.2350	11.4040***

***p < 0.001, **p < 0.01, *p < 0.05.

**Table 4:** Total effect, direct effect, and indirect effect among the variables.

Effect	Effect size	BootSE	BootLLCI	BootULCI	Relative effect size
Total effect	0.2350	0.0210	0.1940	0.2750	
Direct effect	0.1770	0.0210	0.1360	0.2190	75.3191%
Indirect effect	0.0580	0.0090	0.0420	0.0750	24.6809%

**Figure 2:**
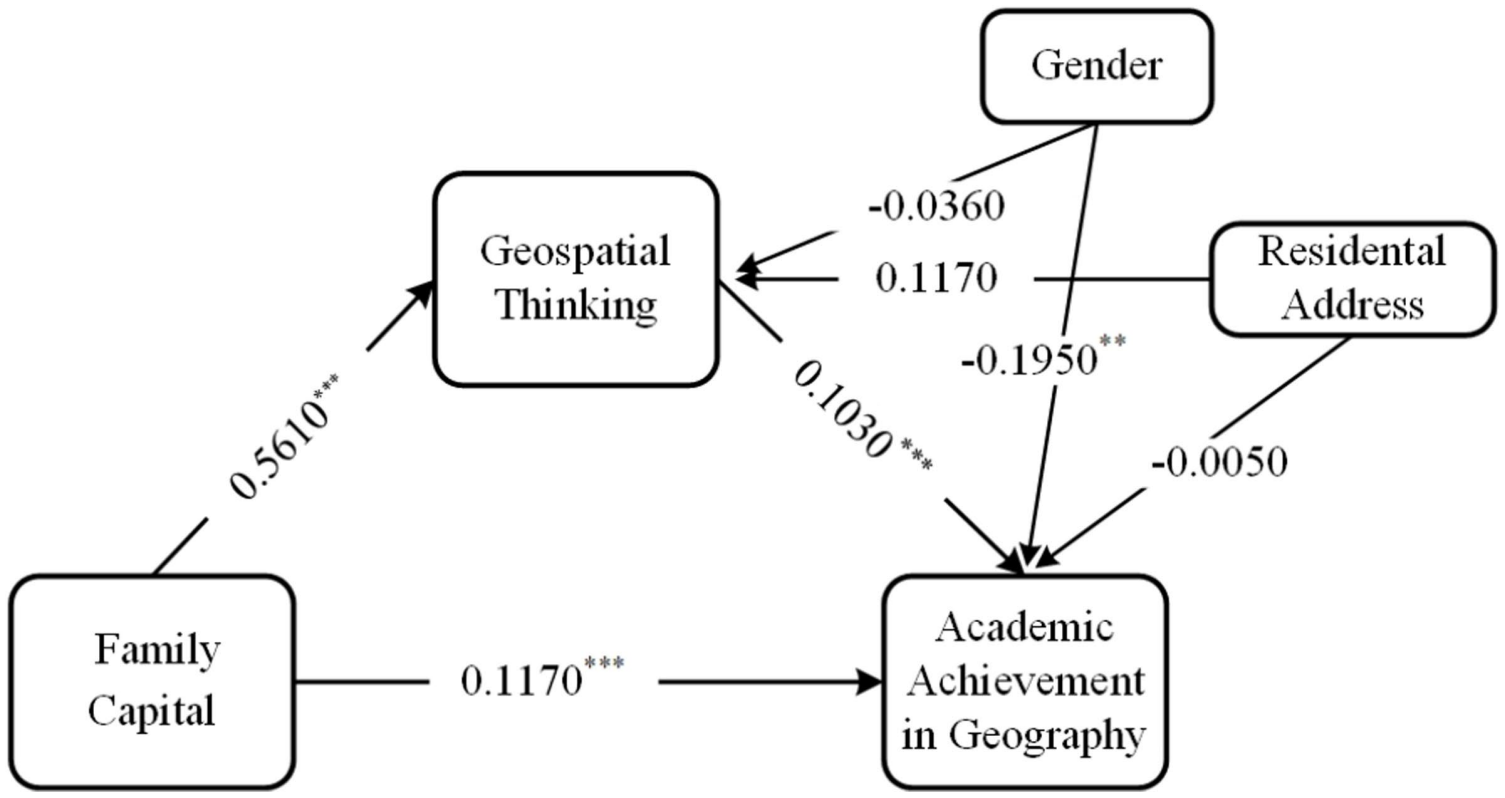
The mediation model showing relationships between Family Capital and Academic Achievement in Geography and the mediating role of Geospatial Thinking.

As can be seen from Table 3, when exploring the relationship between family capital and academic achievement in geography, gender has an impact on academic achievement in geography (*β* = −0.1990, *t* = −2.7190, *p* < 0.01). At the same time, even when geospatial thinking was included in the model, gender still significantly influenced academic achievement in geography (*β* = −0.1950, *t* = −2.7580, *p* < 0.01; Figure 2).

## 5. Discussion

### 5.1. Discussion of the results

In this study, we obtained a mediation model that illustrates the relationship between family capital and academic achievement in geography and the mediating role of geospatial thinking. At the same time, the results of the survey are consistent with the hypotheses of this study and the findings of previous studies.

First, our findings are consistent with Hypothesis 1 and other similar studies. In this study, family capital and academic achievement in geography were positively correlated. This result suggests that good family capital contributes to academic achievement in geography (Bravo Sanzana et al., 2017) and these parents are more likely to focus on providing their children with rich learning opportunities outside the classroom (Conger et al., 2021). For example, Solem et al. (2021) found that academic achievement in geography increased with parental education and the amount of family books, and that the effect size of family books was consistently larger than that of parental education. A possible explanation for this is that children can gain knowledge from the family book collection (Evans et al., 2010), which contributes to higher academic achievement in geography. Meanwhile, children with poorer family capital tend to play closer to home (Ziviani et al., 2008), or more likely to become addicted to the internet or smartphone (Zhang et al., 2018), reducing the effectiveness of geography learning. While children with better family capital are more likely to engage in outdoor activities with nature, which is conducive to their spatial awareness and academic achievement in geographic (Brookfield, 2022; Mason et al., 2022; Pastor et al., 2022). Similar research has shown that higher levels of family capital are associated with academic achievement in geography (Zhang et al., 2022). In addition, the educational level of parents and the socioeconomic status of the family also significantly predict academic achievement in geography (An et al., 2019). Related studies have found that students’ chances of academic success increase when their parents have high levels of literacy (Cheng and Kaplowitz, 2016). Highly educated parents have a great potential to provide their children with a social environment that is beneficial to learning (Taljūnaitė, 2020). At the same time, they understand how to be successful in school (Roosa et al., 2012), which provides an advantage for children to achieve high levels of academic achievement in geography.

Second, the findings are consistent with Hypothesis 2 and other studies indicating that family capital plays a positive predictive role in geospatial thinking. This finding suggests that higher levels of family capital are more conducive to the development of individual thinking (Goudeau et al., 2017), particularly geospatial thinking (Zhang et al., 2022). Studies have shown that there are significant differences in children’s spatial performance even before they enter formal school, and such differences are closely related to parental spatial language and spatial gestures (Clingan-Siverly et al., 2021). It should be noticed that well educated parents use more spatial relational vocabulary and their children will show the greater capability of spatial thinking (Casasola et al., 2020). This is consistent with other similar studies which showed that students from higher income areas had better spatial performance than students from lower-income areas (Casey et al., 2011). Urban students have better educational backgrounds and opportunities, and their levels of geospatial thinking tests are better than rural students (Tomaszewski et al., 2015). Similarly, students from higher socioeconomic status families performed remarkably better in spatial terms than those from lower socioeconomic status families (Carr et al., 2018). The likely explanation is that families with higher economic and literacy levels have a tendency to provide their children with a rich resources and superior learning conditions (Jin et al., 2017) including books, maps, etc. (Bein et al., 2009), to foster their spatial thinking are developed (Zhang et al., 2019).

Third, the findings validated Hypothesis 3 and other relevant studies, showing that geospatial thinking has a positive impact on academic achievement in geography. Similar studies have shown that there are significant differences in academic achievement in geography between students with different spatial thinking, and students with higher spatial thinking were found to have higher academic achievement in geography (Aliman et al., 2019). Meanwhile, students with strong geospatial thinking perform better in geography (Lee and Bednarz, 2009; Klonari and Likouri, 2015; Gold et al., 2018). A possible explanation for this is that geospatial thinking helps to understand and apply geographical knowledge, geographical data (Perugini and Bodzin, 2020). Also, Carbonell-Carrera et al. (2020) demonstrated that good geospatial thinking facilitates students to apply geographic knowledge in solving geographic problems, which is beneficial to their academic achievement in geography (Huynh and Sharpe, 2013). As previously noted, geospatial thinking is considered an important aspect of geography education (Havelková and Hanus, 2021), and has a positive impact on geography learning (Nielsen et al., 2011). As a result, teachers are more willing to use geospatial technology in the geography classroom to strengthen the training of students’ geospatial thinking and further enhance their academic achievement in geography (Hammond et al., 2018).

Fourth, the findings are consistent with Hypothesis 4. We found that geospatial thinking can partially and positively mediate the relationship between family capital and academic achievement in geography. This suggested that family capital not only directly influences academic achievement in geographic, but also influences it indirectly by geospatial thinking (Demetriou et al., 2020), which is in line with similar research findings. Family capital has an impact on children’s cognitive, spatial and psychological development in many ways, and children with low family income have difficulties with language, cognitive development and spatial characteristics (Ip et al., 2016; Zhang et al., 2019). Similar studies have shown that children at the age of three are already map readers (Blaut et al., 1970), and this is inextricably linked to the influence of the home environment (Uhlenberg and Geiken, 2021). Meanwhile, children’s cognitive and spatial thinking can influence academic achievement in geography (Wang et al., 2013). Research showed that family socioeconomic environment affects children’s ability to apply basic cognitive skills such as spatial reasoning to academic performance (Casey et al., 2011). Compared with children from wealthier families, less privileged children have less access to spatially stimulating objects and resources (e.g., blocks, puzzles, maps, etc.) (Dearing and Taylor, 2007). These items are effective in enhancing individuals’ geospatial thinking (Collins, 2018), so disadvantaged family capital is detrimental to the acquisition of academic achievement in geography. In conclusion, good family capital contributes to the development of geospatial thinking and related cognitive skills, which have a positive impact on academic achievement in geography.

In this study, geospatial thinking only partially mediates the relationship between family capital and academic achievement in geography. Analysis of the data showed that family capital had the greatest impact on academic achievement in geography in the model (75.3191%), with geospatial thinking playing only a partially mediating role (24.6809%). In other words, when geospatial thinking are weak, it is still possible for higher family capital to improve students’ academic achievement in geography. Also, students coming from families with lower family capital can also expect some improvement to their academic achievement in geography by enhancing their geospatial thinking.

### 5.2. Implications

This study provides a new perspective for the study of family capital, geospatial thinking and academic achievement in geography, and has important theoretical and practical significance. Theoretically, this study links family capital with academic achievement in geography, explores the important mediating role of family capital in geospatial thinking, and deepens the research on the impact of family capital on academic achievement in geography. The mediating role of geospatial thinking further uncovers the mechanisms underlying the academic achievement in geography. Students from better family environments are more likely to get a better geospatial thinking and academic achievement in geography, as well as geospatial thinking promotes the development of academic achievement in geography. Therefore，it is necessary to focus on both students’ family capital and geospatial thinking in the process of geography learning, and carry out more geospatial thinking training to improve academic achievement in geography。.

### 5.3. Limitations and future directions

There are some limitations to this study. First, it has a cross-sectional design. Second, the participants were all from a particular region in western China, which may affect the generalizability of the study results. Future researchers could carry out longitudinal surveys to gather relevant data over a period of time, or conduct in-depth surveys of students from different areas. Furthermore, it is possible to explore which dimension of geospatial thinking mediates the relationship between family capital and academic achievement in geography. Finally, by analyzing the mechanisms underlying the influence of family capital on geospatial thinking and academic achievement in geography, we can provide a direction for future research on how individuals with disadvantaged family capital improve their academic achievement in geography and geospatial thinking, and effectively help less-privileged students to achieve higher levels of academic achievement in geography.

## 6. Conclusion

This study explored the relationship between family capital and academic achievement in geography, and the mediating role of geospatial thinking between the two. The results showed that upper-secondary-school students with better family capital had higher academic achievement in geography. In addition, upper-secondary-school students with stronger geospatial thinking will have higher academic achievement in geography than those who are poorer at it. It is worth noting that despite the mediating role of geospatial thinking, differences in students’ academic achievement in geography are still largely influenced by family capital.

## Data availability statement

The raw data supporting the conclusions of this article will be made available by the authors, without undue reservation.

## Ethics statement

The studies involving human participants were reviewed and approved by Ethics Committee of Zhejiang Normal University. Written informed consent to participate in this study was provided by the participants’ legal guardian/next of kin.

## Author contributions

JG and JZ designed the research. JZ, TS, XL, YX, ZW, and YY carried out the literature search and data analysis. JZ, TS, XL, YX, ZW, YY, and JG wrote the paper. All authors have read and agreed to the submitted version of the manuscript. All authors contributed to the article and approved the submitted version.

## Funding

This research was funded by the National Office for Education Science Planning, grant number BAA180017.

## Conflict of interest

The authors declare that the research was conducted in the absence of any commercial or financial relationships that could be construed as a potential conflict of interest.

## Publisher’s note

All claims expressed in this article are solely those of the authors and do not necessarily represent those of their affiliated organizations, or those of the publisher, the editors and the reviewers. Any product that may be evaluated in this article, or claim that may be made by its manufacturer, is not guaranteed or endorsed by the publisher.

## Footnotes

^1^ https://www.oecd.org/pisa/data/2018database/

^2^ https://people.rit.edu/~bmtski/rw_stat/STAT_baseline_July_2013.pdf
